# Identification of key regulators in parathyroid adenoma using an integrative gene network analysis

**DOI:** 10.6026/97320630016910

**Published:** 2020-11-30

**Authors:** Nikhat Imam, Aftab Alam, Mohd Faizan Siddiqui, Mohd Murshad Ahmed, Md. Zubbair Malik, Md. Jawed Ikbal Khan, Romana Ishrat

**Affiliations:** 1 Institute of Computer Science and Information Technology, Department of Mathematics, Magadh University, Bodh Gaya-824234, Bihar, India; 2 Centre for Interdisciplinary Research in Basic Sciences, Jamia Millia Islamia, New Delhi-110025, India; 3 International Medical Faculty, Osh State University, Osh City, 723500, Kyrgyz Republic, Kyrgyzstan; 4 School of Computational & Integrative Sciences, Jawaharlal Nehru University, New Delhi, India; 5 Department of Mathematics, Mirza Ghalib College, Magadh University, Bodh Gaya-824234, Bihar, India

**Keywords:** Microarray gene expression, parathyroid adenoma, primary hyperparathyroidism, DEGs, gene ontology, network analysis

## Abstract

Parathyroid adenoma (PA) is marked by a certain benign outgrowth in the surface of parathyroid glands. The transcriptome analysis of parathyroid adenomas can provide a deep insight into actively expressed genes and transcripts. Hence, we analyzed and compared the gene expression profiles of parathyroid adenomas and healthy parathyroid gland tissues from Gene Expression Omnibus (GEO) database. We identified a total of 280 differentially expressed genes (196 up-regulated, 84 down-regulated), which are involved in a wide array of biological processes. We further constructed a gene interaction network and analyzed its topological properties to know the network structure and its hidden mechanism. This will help to understand the molecular mechanisms underlying parathyroid adenoma development. We thus identified 13 key regulators (PRPF19, SMC3, POSTN, SNIP1, EBF1, MEIS2, PAX9, SCUBE2, WNT4, ARHGAP10, DOCK5, CAV1 and VSIR), which are deep-rooted from top to bottom in the gene interaction network forming a backbone for the network. The structural features of the network are probably maintained by crosstalk between important genes within the network along with associated functional modules.Thus, gene-expression profiling and network approach could be used to provide an independent platform to glen insights from available clinical data.

## Background

Parathyroid adenoma (P.A) is marked by a certain benign tumour parathyroid gland. Parathyroid glands are four in Number and very small in size, are positioned nearby the backside of the thyroid gland and release parathyroid hormone (PTH). Parathyroid hormones help to regulate the quantity of calcium (Ca) and phosphorus (P) in the blood. The Parathyroid adenoma outgrowth causes the release of more PTH in the affected gland that it normally should, lead to disruption in calcium and phosphorous levels in the blood. This condition is called hyperparathyroidism [1]. The Parathyroid adenoma (PA) is solely responsible for 80%-85% of hyperparathyroidism [2], but other causes of primary hyperparathyroidism include parathyroid hyperplasia diffuse (10%-12%), multiple parathyroid adenomas (4%-10%) and, rarely occurring parathyroid carcinoma (1%). Adenomas are most common in any individuals of 50 to 70 years old; however, they can occur at any age. Women are more affected by 3-times as often as men [3,4]. Almost everyone who inherited MEN syndromes developed increased activity of the parathyroid glands (hyperparathyroidism) that develop non-cancerous (benign) tumours. The MEN syndrome has three famous forms, i.e. MEN 1, MEN 2A, and MEN 2B and the currently reported form is MEN4. In this syndrome, age has no bar, and it occurs in both men and women [5]. The tumour-suppressor gene MEN1 and proto-oncogene CCND1 are well known key driver genes for sporadic parathyroid adenoma. Recent studies suggest that mutation in MEN1, POT1 and RAP1B can also cause parathyroid adenomas [7- 9]. In the past, many studies had been done to study the benign tumours in the parathyroid glands and compare the genetic profiling of parathyroid adenomas and healthy parathyroid tissues [10-21]. However, still, its pathogenesis is largely unknown. Thus, elucidation of the genetics of parathyroid adenoma would be of great interest and may explain the formation of parathyroid adenomas as well as aid the diagnosis of these tumours. Therefore, it is of interest to compare the gene expression profiles of parathyroid adenomas, and healthy parathyroid gland tissues from Gene Expression Omnibus (GEO) is a database [22] followed by network approach which gave us the base to distinguishing the potential pathways and key regulatory genes that may take part in the development of parathyroid adenoma.

## Materials and Methods:

**Figure 1:**
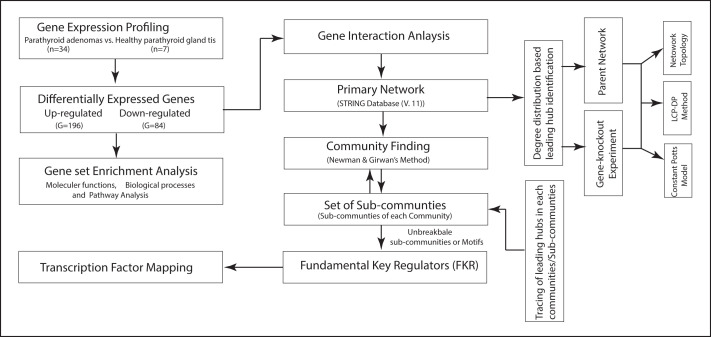
Schematic representation of the workflow.

The detailed workflow of the study depicted in (Figure 1).

## Inclusion Criteria for Differentially Expressed Genes:

In our study, we did a concurrent genome-wide analysis of GSE83421 (Platform: GPL22020) [23] and GSE10317 (Platform: GPL570) in parathyroid adenoma patients selected from Gene Expression Omnibus (GEO) database[24]. A total of 33 parathyroid samples taken from the above GSE series, were categorized into two groups, e.g. normal parathyroid (n=7) and parathyroid adenoma (n=26). In our sample, we excluded all the factors like gender, age etc. These comparisons between normal and adenoma condition shows genes that might be involved in hyperproliferation and PTH hypersecretion in tumours. The background data correction and normalization were performed by platform-specific normalisation in R using the packages limma [25] for Oligo dataset and Affy (RMA) for Affymetrix datasets. All data were quantile normalized, and log2 transformed, we selected only those probes as the DEGs whose expression value changed by at least 1.5 fold change (logFC ≥1.5) as compared with healthy controls and adjusted P-value < 0.05 were set as the cutoff criterion. The probe IDs were changed into their respective gene symbols using the online server Synergizer [26]. By integrating these expressions profiling data, we recognized 280 genes that are differentially expressed in adenoma tissues that may serve as a prognostic molecular signature for parathyroid adenoma.

## Classification, GO and Pathway Enrichment Analysis:

All the differentially expressed genes (DEGs) were classified by various protein classes using PANTHER v.13.0 [27]. Moreover, the functional analysis of all DEGs was done using a functional annotation tool, e.g. DAVID (http://david.abcc.ncifcrf.gov/) [28], which unriddle the biological sense behind the query gene list. Further, GO associations of identified DEGs were analyzed by molecular functions (MF), biological process (BP) and identify biological pathways enriched among those DEGs.

## Networks Construction and Characterization of Topological Properties:

The identified differentially expressed genes (DEGs) were used to build the gene interaction network. The STRING.v.11 (Search Tool for the Retrieval of Interacting Genes/Proteins) was used to construct gene interaction network (medium confidence score < 0.5) [29] and then visualized in Cytoscape v3.7. The Cytoscape is a network visualization platform which supports a large range of plug-ins relative to network construction and its analysis [30]. The graph union operation was used in network construction, and the duplicate edges and self-loops were removed. In the network, each node represents the gene or gene product, and edges represent the connection between the nodes.

Moreover, network analyser was employed for the calculation of basic network metrics, including the number of nodes associated with a particular number of edges, degree exponent, degree distribution, path length and clustering coefficient, leading hubs etc. The complex network's structural properties are distinguished through the nature of the topological variables. There are several centrality measures with different usage and level of information [31], but most of the centrality measures are less common. So, our study is based on only four important centrality measures named Degree centrality, Closeness centrality, Betweenness centrality and Eigenvector centrality, which are generally used to analyze the PPI networks. Since all the centrality measurements (centrality scores) were calculated using CytoNCA [32] for each node in the network. The details of network topological properties and centrality measures were already given in our previous article by Aftab et al. [33].

## Community Detection in Network and Analysis:

In the hierarchical network, to determine the behaviour of modular and its properties; It is crucial to know about the activities of the network at different levels of hierarchy and to know the principle on which the network is self-organized. In this study, the Leading Eigen Vector method (LEV) [33] was employed to identify the communities in the main network using 'igraph' package in R [36]. For community detection, Leading Eigenvector (LEV) is the most promising method, as it measures the eigenvalue epitomizing the importance of the individual link. To grub only motif in the network, first, we identified modules and followed up by sub-modules at each level of organization.

## Gene-tracing in Network and Analysis:

The tracing of genes from the primary network to motif level was performed based on the representation of the particular genes in various sub-modules. Finally, these genes (hub-nodes) which were present in the modules at each hierarchical level were considered as the key regulators [37]. These key regulators are deep-rooted in the network and give the strength to the network to maintain its assortativity.

## Hamiltonian Energy (HE) Calculation:

The organisation of the network at various levels by using the Constant Potts Model [38] was done by calculating the Hamiltonian energy (HE) of the network.

## Transcription Factor and Target Gene Screening:

In our study, we have screened the major transcription factor of our target genes using the TRRUST (v2.0) [40]. It contains 8,444 TF-target regulatory relationships of 800 humans. All the information in the database is taken from 11,237 PubMed articles, which define small-scale experimental studies of transcriptional regulations.

## Results and Discussion:

### Classification of Differentially Expressed Genes (DEGs):

**Figure 2:**
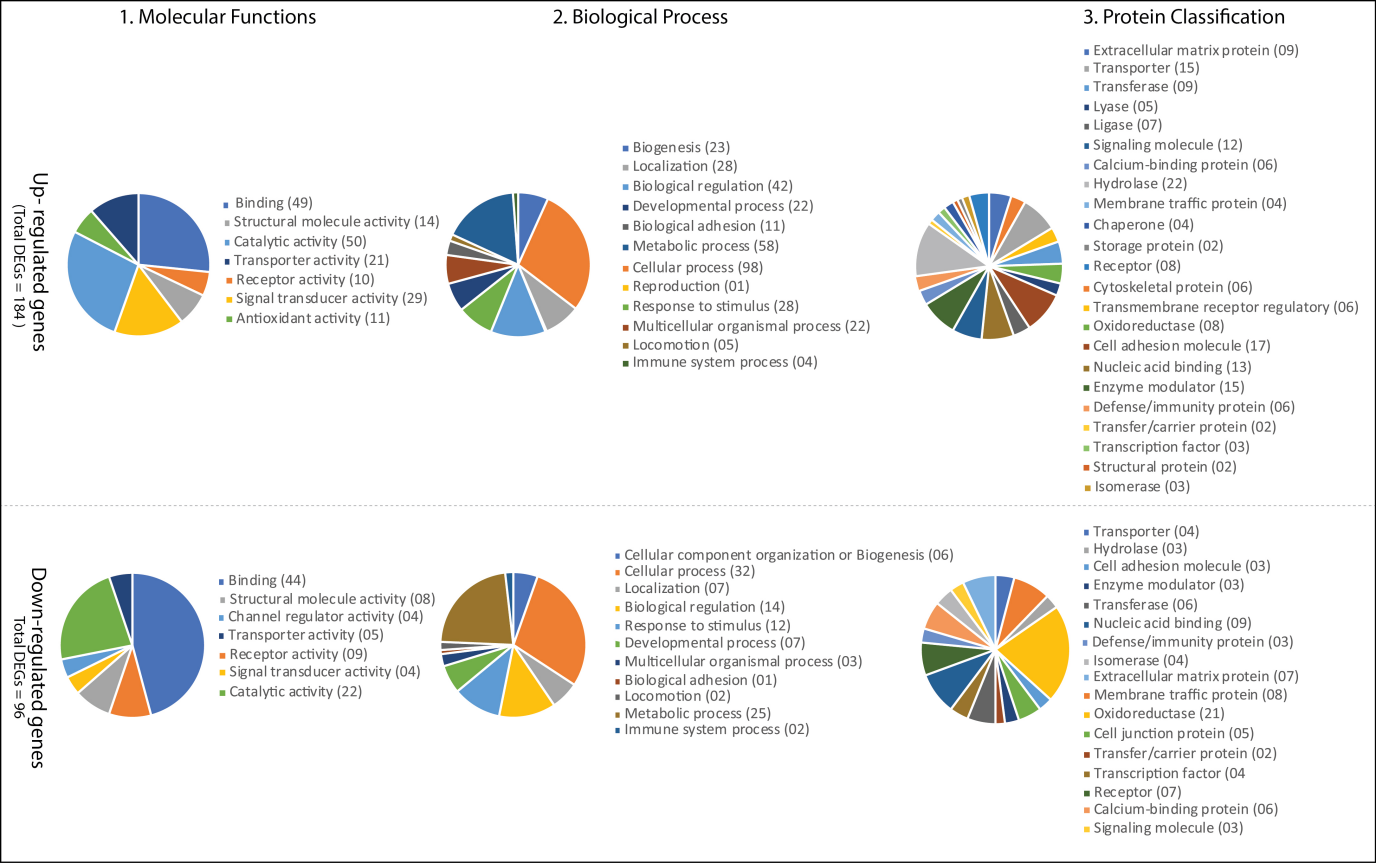
Functional Classification of Differentially expressed genes associated with parathyroid adenoma according to (A) Molecular function, (B) Biological process and (C) protein classes.

A total of 280 differentially expressed genes (DEGs) were identified from three GSE series after an extensive comparison of parathyroid adenoma with normal parathyroid tissues. Out of 280 DEGs, 196 genes were up regulated, and 84 genes were down regulated. Next, all the DEGs were grouped according to 'molecular function', 'biological process' and 'protein classes'. All these predicted DEGs showed a broad spectrum of molecular functions for up-regulated DEGs, which are involved in a wide array of processes like binding proteins (RNA and DNA), catalytic activity, and transporter activity. Similarly, for down-regulated genes, the main molecular functions are binding proteins and catalytic activity. The 02 most abundant GO-biological process groups for up and down-regulated genes are -" Cellular Process" and " Metabolic Process" which is not astonishing as these genes are required in the most basic life processes. The "Cellular Process” includes cell cycle, cell to cell signalling, cell component movement, proliferation, growth and cytokinesis. The "Metabolic Process" includes metabolisms of carbohydrate, lipid, nucleobase-containing compound, protein and cellular amino acid. These DEGs presented a broad range of protein classes for up-regulated genes, which are involved in a wide array of processes such as hydrolase (a sub-category of Proteases and Phosphatases), cell adhesion molecules, enzyme modulator and transporter. In the case of down-regulated genes, the main protein classes are oxidoreductase, nucleic acid binding protein, membrane traffic protein, receptor and extracellular matrix protein. The detailed results are depicted in (Figure 2).

### Network Construction (‘Hierarchical Scale-free Network'):

**Figure 3:**
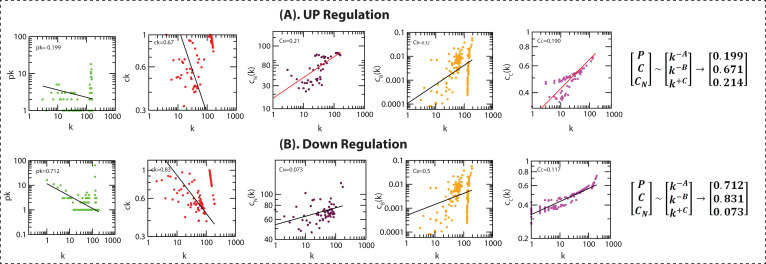
Topological properties of the networks: The behaviours of degree distributions (P(k)), clustering coefficient (C(k)), Neighbourhood connectivity (CN(k)), betweenness (CB(k)) and closeness (CC(k)) measurements as a function of degree k. The lines are fitted lines with power laws in the data sets.

The identified genes (280 DEGs) were used to build a gene interaction network. We built two networks for up-regulated and down-regulated genes individually. The up-regulated network consists of 217 nodes and 10973 edges with having cluster coefficient value 0.802, while Down-regulated network comprising 290 nodes and 11272 edges with network cluster coefficient value is 0.739. The clustering coefficient (measure from 0 to 1) of the network is used to check the network density. The topological variables of the network obeyed power-law distributions. The probability of 'clustering coefficient C(k)', 'degree distributions P(k)', and 'Neighbourhood connectivity CN(k)' showed the fractal nature of the network (Figure 3). Thus, the topological network properties showed the same self-organized behaviour into a scale-free manner and have hierarchical properties.

### Find out key regulatory genes and their properties:

**Figure 4:**
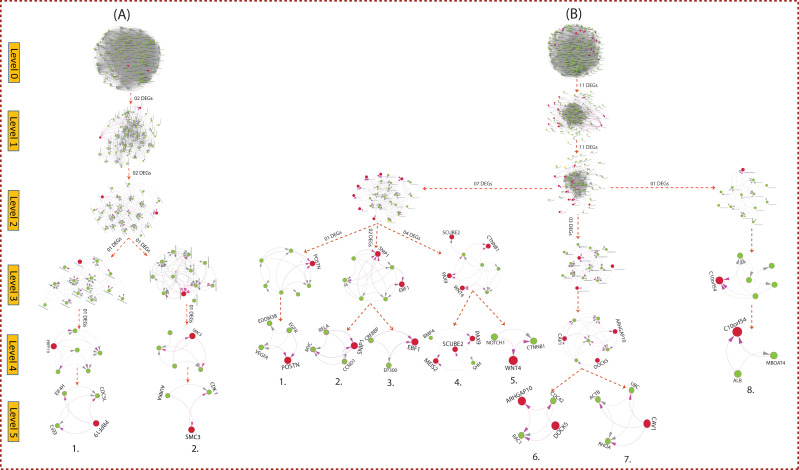
Network/modules/sub-modules at different network levels, which accommodate leading hubs and key regulators.

**Figure 5:**
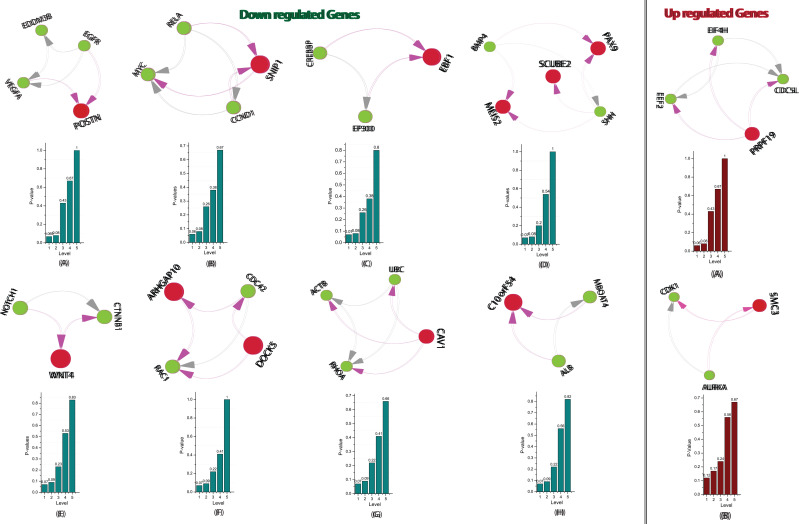
This figure shown the fundamental key regulator obtained from the main networks to motif/hub through various modules/sub-modules at various level of organization. The probability distribution of the 13 fundamental key regulators (11 Down and 02 Up-regulated) as a function of the level of organization is shown.

**Figure 6:**
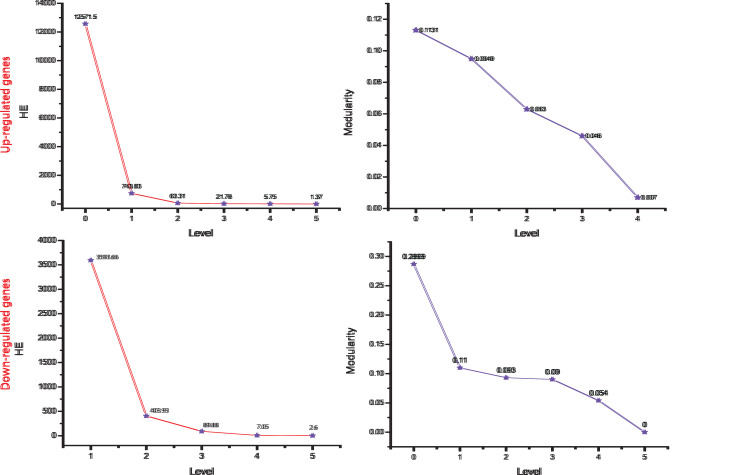
Energy and modularity distribution in main networks quantified by Hamiltonian (HE) and Modularity calculation as a function of network levels.

In PTA network, 13 genes (02 Up and 11 Down-regulated genes) are found to be fundamental key regulators (FKR), which maintain a low profile. Since the leading hub's popularity gets transformed with its activities and regulating mechanisms, we can't say that all the main hub genes work as key regulators for clinical and drug target genes. However, few of them called as fundamental key regulators (FKR) can be significant and can be elucidated as deeply rooted hub genes in the main network, which can be reached from top to bottom level ( at motif level ) via various levels of the organization. The arrangement of the modular structure at different levels of the organization is performed through N & G's standard community finding algorithm. Our networks are found to be hierarchically organized through various levels (Figure 4). We identified a total of 10 hubs that contains 13 key genes (02 Up and 11 Down-regulated) in the networks, shown in (Figure 5). We found that the modularity (QN) and Hamiltonian energy (HE) declines as one goes from top to down in the networks (Figure 6).

### Transcription factor mapping:

**Figure 7:**
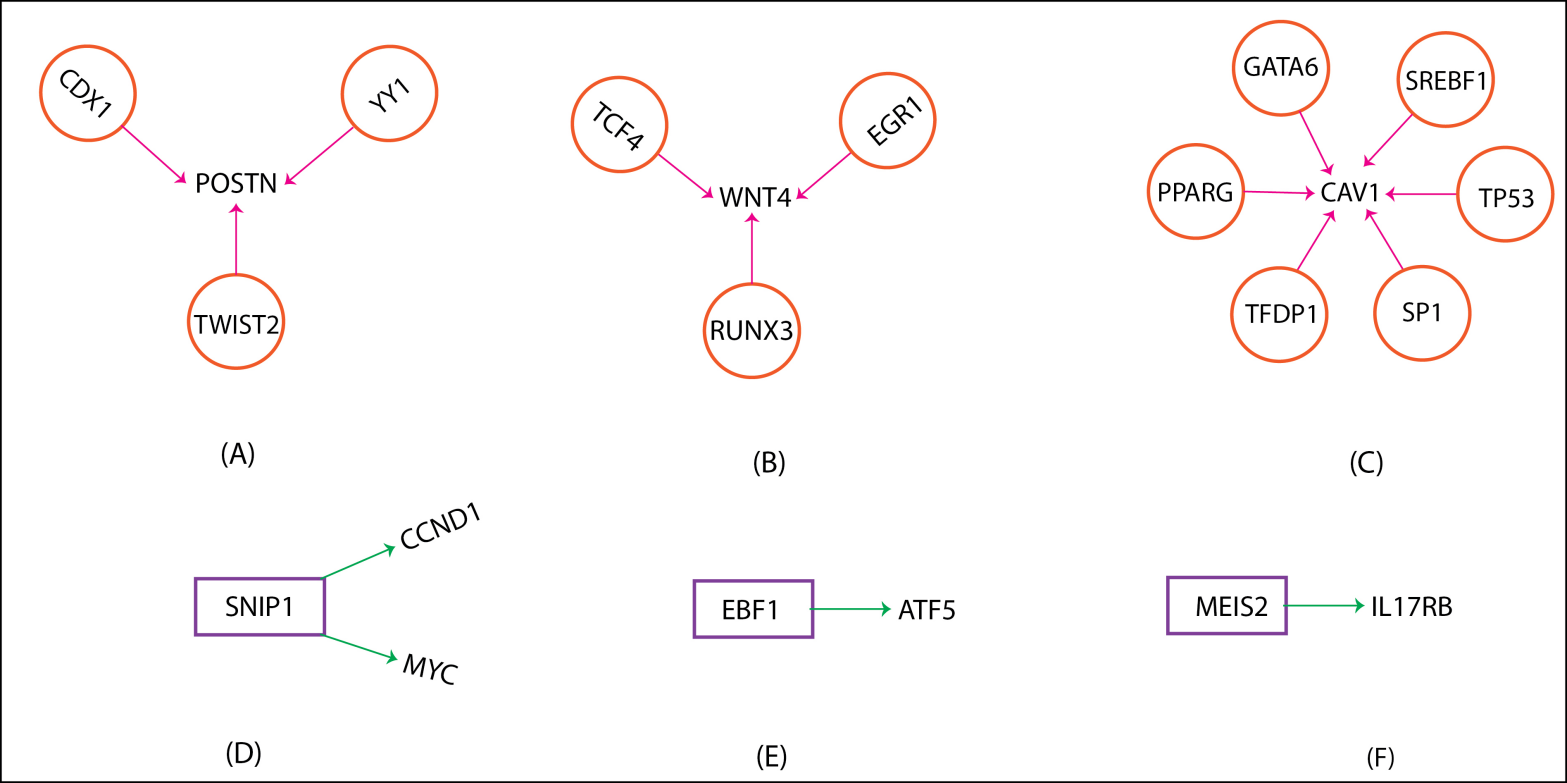
The genes POSTN, WNT4 and CAV1, are regulated by 12 transcription factors (CDX1, TWIST2 & YY1; TCF4, EGR1 & RUNX3; GATA6, SREBF1, TP53, SP1, TFDP1 & PPARG respectively) and three genes namely SNIP1, EBF1 and MEIS2 are working as the transcription factors and regulated many target genes in human.

We identified 12 transcription factors with P_value < 0.01, that regulated our three target genes (POSTN, WNT4 and CAV1), and 03 genes (SNIP1, EBF1 and MEIS2) working as a transcription factor (TF) that regulate the several target genes via activation/repression/another mode of action in the human, shown in (Figure 7). The information about the rest of the genes (PRPF19, SMC3, PAX9, SCUBE2, ARHGAP10, DOCK5 and VSIR) is still unknown.

Primary hyperparathyroidism (PHPT) is a well-known cause of hypercalcemia in ambulatory patients & is characterized by excessive secretion of the parathyroid hormone (PTH). The parathyroid adenoma is the underlying cause in 85% of the cases. However, parathyroid malignancies occur in <0.5% of all cases [41] and usually, they are rare. About 75% of 80% of PHPT patients are asymptomatic. Its clinical manifestations are secondary to hypercalcemia, which include overt bone disease, renal calculi, nonspecific gastrointestinal symptoms, as well as cardiovascular and neuromuscular dysfunction [42]. Gene expression profiling method has established an absolute difference between the transcriptome of normal parathyroid & adenoma tissues and revealed significant genetic, molecular markers, which can create a basis for initial diagnosis and encourage personalized medication.

**Figure 8:**
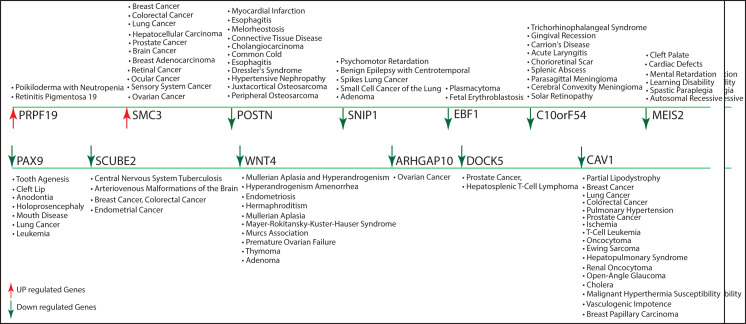
Representation of 13 key regulators which are involved directly or indirectly in parathyroid adenoma but also in various types of cancers and other life-threatening diseases in human.

The knowledge of the regulation of disease-network, in the field of Pharmacogenomics, has great application in the drug discovery. In the present study, we have emphasized on network-regulated genes. The network of classified genes from parathyroid adenoma displays hierarchical characteristics. This indicates that the networks are segregated at the organizational level and include interconnected modules/sub-modules. The hierarchical nature of the network is visible during synchronization, as it confirms numerous significant functional regulations of the network. Individual gene-activities in the process assume less importance. In our networks, a total of 13 key regulators (PRPF19↑, SMC3↑, POSTN↓, SNIP1↓, EBF1↓, MEIS2↓, PAX9↓, SCUBE2↓, WNT4↓, ARHGAP10↓, DOCK5↓, CAV1↓ and VSIR↓) were recognised by affecting motifs and module regulation, representing their biological significance, their role in the basis of network activities and associated regulations, and how they could be a most likely target gene of disease. We also identified the biological activity and pathways in which these key regulator genes are involved, as shown in (Suppli_data 1). These genes are also involved in many life-threatening diseases including various types of cancers, tumours, Spinocerebellar ataxia, Alzheimer, Asthma, Diabetes, Vascular disease, Esophagitis, Inflammatory-Bowel disease, Plasma cytoma, Pulmonary hypertension, Tooth agenesis, Leukemia among others. The complete details are given in (Figure 8).

Significantly, two up-regulated genes in the adenoma specimens are PRPF19 (Pre-mRNA-processing factor-19) and SMC3 (Structural maintenance of chromosomes protein 3). PRPF19 plays an important role in the spliceosome machinery of humans. It has been seen that this DNA repair gene is over expressed in tumours [43]. The gene SMC3 is a component of a number of nuclear multimeric protein complexes that are required in DNA recombination & repair and chromosomal segregation. Activation of the tumorigenic cascade is done by Up-regulation of SMC3 [44]. So, these both genes may also contribute to the development of parathyroid adenoma. Besides, A total of 11 genes found to be down regulated in our study. A previous study has suggested that the down-regulation of SCUBE2 is significantly associated with the depth of tumour invasion, distant metastasis and lymph node metastasis [45]. Thus, SCUBE2 gene may work as a novel tumour suppressor and a possible target for parathyroid adenoma patient. The study suggests that ARHGAP10 acts as a tumour suppressor by affecting metastasis and Wnt signalling pathways. The Wnt pathway plays various roles in the progression, metastasis and initiation of various types of tumour and cancers. It has been reported that the expression of ARHGAP10 decreases within the tumour tissue [46]. So, down regulation of ARHGAP10 may help in adenoma progression. Moreover, the gene POSTN interacts with several signalling cascades and has several functions in inflammatory diseases and tumours to modulate the gene expression. In our study, we found that POSTN is downregulated in parathyroid adenoma tissue, but the exact mechanism about how its downregulation helps in adenoma growth remains unclear. But, it has been reported that POSTN is down-regulated in high grade human bladder cancers [47]. It has been already reported that the down-regulation of CAV1 may contribute to adenomatous PT cell proliferation and function. The gene CAV1 is a potent negative regulator of a variety of mitogenic signalling pathways. The downregulation of CAV1 in parathyroid tissues may help to enhance the activity of cyclin D1 that plays a role in the pathogenesis of a much larger proportion of parathyroid adenomas [48]. It has been shown that SNIP1 plays a vital role in co-or post-transcriptional Cyclin D1 mRNA stability. H342 rapidly reduce the SNIP1 expression and consequently, causes downregulation of c-Myc target genes. These changes in gene expression are induced by H342 that may lead to mitochondrial dysfunction, and this dysfunction may contribute to tumorigenesis [49]. The role of EBF3 gene is still unclear that how it is involved in adenoma growth, but functional studies suggested that EBF3 activate the genes which are involved in apoptosis and cell cycle arrest while repressing genes involved in cell survival and proliferation and that EBF (transcription factors) serve as tumour suppressors in various cancers [50]. So, the downregulation of EBF3 (tumour suppressors gene) may also be crucial for adenoma development.

The WNT4 gene family contains structurally associated genes, which encode secreted signalling proteins. It has been reported that Wnt expression is reduced in endometrial carcinomas and in invasive ductal breast carcinomas [51]. The downregulation of WNT4 is expected to alter the Wnt signalling pathway that may disrupt the balance of cell proliferation and apoptosis that leads to adenoma. The gene MEIS2 is a transcriptional factor that regulates the expression of large sets of downstream target genes by direct binding to their promoter DNA. The MEIS2 controls cell growth and differentiation during embryogenesis and carcinogenesis. A recent study shows that the down-regulation of MEIS2 can increase tumour growth over time [52]. The DOCK5 is a large protein that participated in intracellular signalling networks. Previous studies suggest that regulation of DOCK5 decreases in various types of tumours [53]. The C10orF54 (VSIR or VISTA) is a type I transmembrane protein that functions as an immune checkpoint. The VISTA expression on parathyroid adenoma and its related regulatory mechanisms is still not clear. So, more study is needed to elucidate the role of VSIR in adenoma growth [54]. The PAX9 gene works as a transcription factor of the PAX family. It has been shown that the expression of PAX9 is downregulated in most of the invasive carcinomas and epithelial dysplasias but PAX9 expression levels may vary in different tumours and may play different roles via different mechanisms in various tumours. So, downregulation of PAX9 may put an impact on the cell cycle by maintaining tumour cells [55-57].

In addition, we used a reliable computational approach to find the transcription factors (TF) that may play an important role under a certain biological condition. The TRRUST server was used to identify transcription factors of our target genes. Many of these key factors are closely associated with the human immune system. The POSTN gene is regulated by three target genes, namely: (i) CDX1-it is associated with caudal-related homeobox transcription factor family and has a direct regulation between CDX1-binding and the expression level of POSTN [58]. (ii) POSTN is transactivated by TWIST2 [59] and (iii) YY1 has a functional role in transcription control over the human POSTN gene [60]. Similarly, WNT4 is also regulated by three genes: (i) The promotor activity of the WNT4 gene is mutually down-regulated by EGR1 and MM1 protein [61]. (ii) RUNX3 is a Wnt inhibitor, which forms a ternary complex with TCF4 to impaired Wnt signalling, which regulates cell proliferation, apoptosis, and invasion [62]. The EGR1 regulates Wnt through up-regulation of TCF4, which induces stem cell marker LGR5 [63]. The CAV1 (Caveolin-1) gene is regulated by six target genes, namely: (i) it was noticed that the transcription factor GATA-6 binds this promoter, resulting in reduced expression of caveolin-1[64]. (ii) PPARG inhibits CAV1 by prompting its lysosomal degradation, without affecting the mRNA level [65]. (iii-vi) Transcription factors SP1 and TFDP1 are bound to the sites of the caveolin promoter DNA sequence (at 151 to 138 bp) in a complex stabilized form by tumour suppressor protein p53. (v) SREBP1 is a transcriptional regulator of CAV1 expression in response to free cholesterol and mediated its effect via the same E2F/Sp1 site. (vi) The caveolin gene transcription, cell cholesterol, and growth are regulated by TP53 by a novel mechanism [66].

Moreover, we found 03 genes (SNIP1, EBF1 and MEIS2) worked as a transcription factor, which regulates many target genes in humans via activation/repressions and other modes of action. The SNIP1 gene is associated with c-Myc, which is a key regulator of cell proliferation and transformation. SNIP1 increases the transcriptional activity of c-Myc both by stabilizing it against proteasomal degradation and by bridging the c-Myc/p300 complex [67] and SNIP1 also regulates the CCND1 (Cyclin D1) expression and promoter activity [68]. EBF1 is the transcription factor which binds to the ATF5 promoter and regulates the ATF5 transcription in an EBF-binding site in an independent manner [69]. MEIS2 is a homeobox transcription factor and known as an endogenous substrate of IL17RB (CRL4) and inhibits the function of CRBN (a substrate receptor of the CRL4 E3 ligase) in myeloma cells [70].

## Conclusion

We report 13 key regulators (PRPF19, SMC3, POSTN, SNIP1, EBF1, MEIS2, PAX9, SCUBE2, WNT4, ARHGAP10, DOCK5, CAV1 and VSIR), which are deep-rooted from top to bottom in the gene interaction network forming a backbone for the network linked with PA. The structural features of the network are probably maintained by crosstalk between important genes within the network along with associated functional modules. Thus, gene-expression profiling and network approach could be used to provide an independent platform to glen insights from available clinical data in the context of Parathyroid adenoma.

## References

[1] Bilezikian JP (2018). Lancet Lond Engl.

[2] Colognesi A (2006). Minerva chirurgica.

[3] Wolfe SA (2018). Stat Pearls Publishing.

[4] Edafe O (2018). Annals of the Royal College of Surgeons of England.

[5] de Laat JM (2008). Frontiers in Endocrinology.

[6] Khatami F, Tavangar SY (2018). Biomarker Insights.

[7] Arvai Kristof (2012). Pathology oncology research.

[8] Kyle Cromer M (2012). The Journal of clinical endocrinology and metabolism.

[9] Newey PJ (2012). The Journal of clinical endocrinology and metabolism.

[10] Stefaniak, Boguslaw (2003). Nuclear medicine review Central & Eastern Europe.

[11] Chai Young Jun (2019). Journal of clinical medicine.

[12] Schneider Ralph (2015). World journal of surgery.

[13] Shiau YC (2002). Nucl Med Biol.

[14] Hadar Tuvia (2005). Pathology oncology research.

[15] Sayar H (2014). Indian J Pathol Microbiol.

[16] Varshney Shweta (2013). Endocrine.

[17] Kao A (2002). Eur J Nucl Med Mol Imaging.

[18] Kaneko C (1999). Calcified tissue international.

[19] Wang W (1996). APMIS.

[20] Sakaguchi K (1999). Oncogene.

[21] Ludwig L (1999). Endocr.

[22] Clough E (2016). Methods in molecular biology.

[23] Balenga N (2017). J Bone Miner Res Off J Am Soc Bone Miner Res.

[24] Barrett T (2005). Nucleic Acids Res.

[25] Smyth GK (2004). Stat Appl Genet Mol Biol.

[26] Berriz GF (2008). Bioinforma Oxf Engl.

[27] Mi H (2013). Nucleic Acids Res.

[28] Huang DW (2009). Nat Protoc.

[29] Szklarczyk D (2017). Nucleic Acids Res.

[30] Shannon P (2003). Genome Res.

[31] Ashtiani M (2018). BMC Syst Biol.

[32] Tang Y (2015). Biosystems.

[33] Alam A (2019). Front Genet.

[34] Newman MEJ (2006). Phys Rev E.

[35] Newman MEJ (2006). Proc Natl Acad Sci.

[36] Gabor Csardi (2006). Inter J Comp Syst.

[37] Malik MDZ (2019). BMC Cancer.

[38] Traag VA (2011). Phys Rev E.

[39] Traag VA (2013). Sci Rep.

[40] Han H (2018). Nucleic Acids Res.

[41] Kearns AE (2002). Mayo Clin Proc.

[42] Bilezikian IP (2002). J Clin Endocrinol Metab.

[43] Roe OD (2012). PLoS ONE.

[44] Ghiselli G (2006). Mol Cancer.

[45] Song O (2015). Oncol Rep.

[46] Teng JP (2017). Oncol Lett.

[47] Kim CJ (2005). Int J Cancer.

[48] Kifor O (2003). J Clin Endocrinol Metab.

[49] De Araujo LF (2015). Tumour Biol.

[50] Prasad MAJ (2015). Blood.

[51] Jonsson M (2002). Cancer Res.

[52] Bhanvadia RR (2018). Clin Cancer Res.

[53] Spisak S (2012). PLoS ONE.

[54] Lines JL (2014). Cancer Res.

[55] Gerber JK (2002). J Pathol.

[56] Tan B (2017). Mol Med Rep.

[57] Xiong Z (2018). J Pathol.

[58] Xiao SM (2012). Osteoporos Int.

[59] Franco HL (2011). Int J Biochem Cell Biol.

[60] Romeo F (2011). Gene.

[61] Yoshida T (2008). Exp Cell Res.

[62] Liu JB (2011). World J Gastroenterol.

[63] Ernst A (2011). Pathology.

[64] Boopathi E (2011). Am J Pathol.

[65] Yang K (2015). Am J Respir Cell Mol Biol.

[66] Bist A (2000). Biochemistry.

[67] Fujii M (2006). Mol Cell.

[68] Roche KC (2007). Oncogene.

[69] Wei Y (2010). J Biochem.

[70] Fischer ES (2014). Nature.

